# Supplementary material for: COZOID: contact zone identifier for visual analysis of protein-protein interactions
Source: BMC Bioinformatics. 2018 Apr 6;19:125. doi: 10.1186/s12859-018-2113-6 (PMC5889581; doi:10.1186/s12859-018-2113-6)
Supplement: Supplementary file 2 — Software build. Executable binary file of the software tool. (ZIP 118,784 kb) [file 12859_2018_2113_MOESM2_ESM.zip › cozoid/java/docs/beansbinding-1.2.1-doc.zip!/org/jdesktop/el/impl/parser/class-use/ParseException.html]

Uses of Class org.jdesktop.el.impl.parser.ParseException (Beans Binding)


---


|  |  |  |
| --- | --- | --- |
| **Overview** \| **Package** \| **Class** \| **Use** \| **Tree** \| **Deprecated** \| **Index** \| **Help** | |  |
| PREV   NEXT | **FRAMES**    **NO FRAMES**     **All Classes** |  |


---


## **Uses of Class org.jdesktop.el.impl.parser.ParseException**

| Packages that use ParseException | |
| --- | --- |
| **org.jdesktop.el.impl.parser** | Customized version of EL for Beans Binding; not for general use. |

Uses of ParseException in org.jdesktop.el.impl.parser

| Methods in org.jdesktop.el.impl.parser that return ParseException | |
| --- | --- |
| `ParseException` | `ELParser.generateParseException()` |

| Methods in org.jdesktop.el.impl.parser that throw ParseException | |
| --- | --- |
| `void` | `ELParser.And()` |
| `void` | `ELParser.Boolean()` |
| `void` | `ELParser.BracketSuffix()` |
| `void` | `ELParser.Choice()` |
| `void` | `ELParser.Compare()` |
| `AstCompositeExpression` | `ELParser.CompositeExpression()` |
| `void` | `ELParser.DeferredExpression()` |
| `void` | `ELParser.DotSuffix()` |
| `void` | `ELParser.DynamicExpression()` |
| `void` | `ELParser.Equality()` |
| `void` | `ELParser.Expression()` |
| `void` | `ELParser.FloatingPoint()` |
| `void` | `ELParser.Function()` |
| `void` | `ELParser.Identifier()` |
| `void` | `ELParser.Integer()` |
| `void` | `ELParser.Literal()` |
| `void` | `ELParser.LiteralExpression()` |
| `void` | `ELParser.Math()` |
| `void` | `ELParser.Multiplication()` |
| `void` | `ELParser.NonLiteral()` |
| `void` | `ELParser.Null()` |
| `void` | `ELParser.Or()` |
| `void` | `ELParser.String()` |
| `void` | `ELParser.Unary()` |
| `void` | `ELParser.Value()` |
| `void` | `ELParser.ValuePrefix()` |
| `void` | `ELParser.ValueSuffix()` |

---


|  |  |  |
| --- | --- | --- |
| **Overview** \| **Package** \| **Class** \| **Use** \| **Tree** \| **Deprecated** \| **Index** \| **Help** | |  |
| PREV   NEXT | **FRAMES**    **NO FRAMES**     **All Classes** |  |


---
